# The *Babesia* observational antibody (BAOBAB) study: A cross-sectional evaluation of Babesia in two communities in Kilosa district, Tanzania

**DOI:** 10.1371/journal.pntd.0007632

**Published:** 2019-08-14

**Authors:** Evan M. Bloch, Zakayo Mrango, Mabula Kasubi, Jerusha Weaver, Aleksandra Mihailovic, Beatriz Munoz, Anna Weimer, Andrew Levin, Laura Tonnetti, Jeffrey M. Linnen, Vanessa Brès, Douglas E. Norris, Giovanna Carpi, Sheila K. West

**Affiliations:** 1 Department of Pathology, Johns Hopkins School of Medicine, Baltimore, Maryland, United States of America; 2 National Institute for Medical Research, Kilosa, Tanzania; 3 Department of Microbiology, Muhimbili University of Health and Allied Sciences, Dar es Salaam, Tanzania; 4 Dana Center for Preventive Ophthalmology, Johns Hopkins School of Medicine, Baltimore, Maryland, United States of America; 5 Kephera Diagnostics, LLC, Framingham, Massachusetts, United States of America; 6 American Red Cross, Rockville, Maryland, United States of America; 7 Grifols Diagnostic Solutions Inc., San Diego, California, United States of America; 8 Department of Molecular Microbiology and Immunology, Johns Hopkins Bloomberg School of Public Health, Baltimore, Maryland, United States of America; University of Iowa, UNITED STATES

## Abstract

**Background:**

*Babesia*, a tick-borne genus of intraerythrocytic parasites, is understudied in humans outside of established high-endemic areas. There is a paucity of data on *Babesia* in Africa, despite evidence that it is regionally present. A pilot study suggested that *Babesia* was present in a rural district of Tanzania.

**Methodology/Principal findings:**

A cross-sectional study was conducted July-August 2017: residents in a case hamlet that had clustering of subjects with high signal-to-cut off (S/CO) ratios for antibodies against *B*. *microti* in the pilot study, and a control hamlet that had lacked significant signal, were evaluated for *B*. *microti*. Subjects aged ≥15yrs (n = 299) underwent clinical evaluation and household inspections; 10ml whole blood was drawn for *Babesia* transcription mediated amplification (TMA), *B*. *microti* indirect fluorescent antibody testing (IFA) and rapid diagnostic testing (RDT) for *Plasmodium* spp. Subjects aged <15yrs (n = 266) underwent a RDT for *Plasmodium* and assessment by ELISA for *B*. *microti* antibodies. A total of 570 subjects participated (mean age 22 [<1 to 90yrs]) of whom 50.7% were female and 145 (25.5%) subjects were *Plasmodium* RDT positive (+). In those <15yrs, the median ELISA S/CO was 1.11 (IQR 0.80–1.48); the median S/CO in the case (n = 120) and control (n = 146) hamlets was 1.19 (IQR 0.81–1.48) and 1.06 (IQR 0.80–1.50) respectively (p = 0.4). Children ≥5yrs old were more likely to have a higher S/CO ratio than those <5yrs old (p<0.001). One hundred (38%) subjects <15yrs were *Plasmodium* RDT+. The median S/CO ratio (children <15yrs) did not differ by RDT status (p = 0.15). In subjects ≥15yrs, no molecular test was positive for *Babesia*, but four subjects (1.4%) were IFA reactive (two each at titers of 128 and 256).

**Conclusions/Significance:**

The findings offer further support for *Babesia* in rural Tanzania. However, low prevalence of seroreactivity questions its clinical significance.

## Author summary

*Babesia*, a family of tick-borne parasites, causes babesiosis, a disease that is very similar to malaria. *Babesia* species are globally ubiquitous yet understudied in humans outside of a few areas of the world, most notably in the United States. There is very little, published information on *Babesia* in humans in Africa. We conducted a study of two rural communities in Tanzania where earlier findings had suggested *Babesia* was present. Dedicated study teams visited households in the two communities to collect information about the residents’ health as well as factors that could pose risk of exposure to ticks and other infectious diseases. The residents of the two communities also had samples collected for *Babesia* evaluation. The test results revealed that a few of the residents had likely been exposed to *Babesia* in the past but were not actively infected at time of the assessment. The findings provide additional support for *Babesia*’s presence in human populations in Africa. This is important as *Babesia* infection can mimic other infections, notably malaria.

## Introduction

*Babesia* is a ubiquitous [1, 2] genus of intraerythrocytic, apicomplexan parasites, that is increasingly recognized as posing risk to human health. Over 100 species of *Babesia* have been shown to infect vertebrate hosts yet only a few are known to infect humans, of which *Babesia microti* is overwhelmingly representative. *Babesia* is transmitted principally via the ectoparasitism of ixodid ticks. In the case of *B*. *microti*, its principal vector, *Ixodes scapularis* (the black-legged or deer tick), also transmits *Borrelia burgdorferi* (Lyme disease), *Anaplasma phagocytophilum* (human granulocytic anaplasmosis) and *Borrelia miyomotoi* (relapsing fever). Babesiosis, the clinical disease named for infection with any of the *Babesia* species, is frequently uneventful in the immunocompetent human host following a mild, self-limiting or even subclinical course. Symptoms, when they do occur, are those of mild flu-like illness (e.g. fever, myalgia, fatigue, headache and chills). In the case of uncomplicated babesiosis, infection is treatable with a short course of a combination azithromycin and atovaquone [3].

However, *Babesia* poses both diagnostic and clinical challenges. First, typical symptoms and signs of babesiosis are non-specific requiring some level of vigilance for a parasite that has historically been neglected. As such those practicing outside of highly endemic areas may lack awareness of *Babesia*, contributing to delays in diagnosis, with concomitant risk of complicated or severe infection. Second, babesiosis in certain patient subsets, notably those with asplenia, at extremes of age, and/or who are immunocompromised are at high risk for severe infection [3]. *Babesia* is related to *Plasmodium* (malaria) with which it shares pathologic and clinical features. As in the case of malaria, *Babesia*-parasitized red blood cells are subject to hemolysis accounting for clinical complications that include hemolytic anemia, cardiorespiratory/renal failure, disseminated intravascular coagulation and even death [3]. Third, *Babesia* has the ability to establish persistent, asymptomatic infection is some individuals [4]. The mechanism for this is not well understood but, indirectly, poses risk to the blood supply, given that asymptomatic, parasitemic blood donors may unwittingly contribute parasitemic blood to transfusion recipients [5, 6]. *Babesia* is transfusion transmissible via red blood cell containing products. With the exception of the United States where regional *Babesia* screening of blood donors was mandated in 2019, blood donor screening for *Babesia* is not in effect elsewhere in the world. Transfusion recipients are at high risk for severe babesiosis given their overrepresentation of risk factors (e.g. immunosuppression, sickle cell disease etc.). Furthermore, severe anemia is the primary indication for red blood cell transfusions rendering transfusion recipients relatively intolerant of *Babesia*-associated hemolysis, which may account for the high mortality (~20%) reported following transfusion transmitted babesiosis (TTB)[6, 7]. Recognition of risk of TTB in the United States (US) has spurred development of new serological and molecular assays, with a view toward blood donor *Babesia* screening [8–11].

Although the increase in tick-borne and TTB in the US has garnered much attention [12, 13], *Babesia* should be viewed as a global pathogen. Beyond its historical recognition in parts of Europe [14–16], there is a growing number of reports of human babesiosis from areas where *Babesia* has not been well publicized such as in South America [17], Asia [18–21] and Australia [22]. An expanding repertoire of highly sensitive *Babesia* diagnostic assays affords opportunity for global surveillance for this neglected pathogen. This motivated for a pilot study in Africa [23] where, despite a paucity of *Babesia* surveillance data in humans, there was plausible evidence that *Babesia* was present [24–27]. *Babesia’s* presence in ticks and its role as a significant veterinary pathogen in Africa, is well established [24–26, 28–30]. Of particular interest, *B. microti [25]* and *B*. *microti*-like parasites [31] have been recovered from non-human primates in Africa. The pilot study, which evaluated dried blood spots from 1–5 yr old children in Kilosa District, Tanzania, using a *B*. *microti* ELISA assay, demonstrated clustering of individuals with high signal to cutoff (S/CO) ratios in a relatively small number of hamlets and an increase in seroreactivity with age [27]. While suggestive of local exposure, in the absence of confirmatory and ancillary testing, the results were viewed as preliminary. An understanding of *Babesia’s* role in disease in pastoral African communities is important to guide empiric antimicrobial therapy. Furthermore, malaria is widely endemic in Africa: *Babesia* is morphologically similar to *Plasmodium spp*. on microscopy, which could contribute to misdiagnosis and underreporting in those areas where both parasites are encountered, as has been reported, elsewhere [19].

The objective of the *Babesia* Observational Antibody (BAOBAB) study was to determine the prevalence of exposure to *Babesia* in children age <15, using a test for antibodies, and to assess active infection as well as past exposure in adults age 15 years and older. We conducted a whole population screen in two communities. If *Babesia* was present, we sought to gain insight into the risk factors for exposure that might be amenable to intervention.

## Methods

### Ethics statement

Ethical approval for the study was obtained from the Tanzanian National Institute for Medical Research and the Institutional Review Board of the Johns Hopkins School of Medicine. Written informed consent was obtained from all participants. In the case of minors, consent was obtained from guardians and additional assent was obtained in the case of children aged 7-17yrs.

### Setting and population

A cross-sectional study was conducted July-August 2017 in two hamlets in Kilosa District, Tanzania that had participated in the preceding pilot study. The latter was confined to children aged 1–59 months. Residents in a case hamlet (“119”; Kigobele) that had clustering of subjects with high signal-to-cut off (S/Co) ratios for antibodies against *B*. *microti* in the pilot study, and a control hamlet (“483”; Manungu; Kiduhi village) that had lacked significant signal, were evaluated for *B*. *microti*. In addition to clinical evaluation and household inspections, subjects aged ≥15yrs (n = 299) had 10ml whole blood drawn for evaluation by transcription mediated amplification (TMA) for *B*. *microti*, *B*. *divergens*, *B*. *venatorum* and *B*. *duncani*, indirect fluorescent antibody testing for *B*. *microti* and rapid diagnostic testing (RDT) for *Plasmodium* spp. Those aged <15yrs (n = 266) underwent RDT for *Plasmodium* and serological assessment with an ELISA for antibodies to *B*. *microti*. All residents in the participating hamlets were eligible to participate in the study.

### Data collection

A census of each of the houses in the hamlets was conducted prior to sample collection. During the census, a trained field team visited each of the houses and interviewed the head of household. The field team assessed the house for potential risk factors for vector borne illness. These included material composition of the house (i.e. wall, roof and window construction), sleeping conditions (i.e. number of individuals per room, the presence of bed nets, whether sleeping on animal skins vs. a bed) and contact with animals. The perimeter of the house was inspected for proximity to grass and/or an animal pen. If the guardian of children living in the house was present, informed consent was sought, and an invitation extended for a follow-up clinical evaluation and sample collection at a central site in the hamlet.

The household members presented on a designated day for the clinical evaluation and sample collection. This was conducted under full informed consent from adults or legal guardians (in the case of children). Assent was also obtained from minors ≥7yrs old. Explanation was provided in Kiswahili. Maasai was used in hamlet 483 in select cases. The clinical evaluation included enquiry regarding recent symptoms and signs of babesiosis, recent diagnosis and/or treatment for malaria and ongoing antibiotic or antimalarial therapy at time of evaluation. Vital signs were documented.

### Laboratory procedures

Procedures differed by subject age. For subjects aged >1yr but <15yrs, a finger stick was performed on each of the participants and dried blood spot (DBS) collected (i.e. filter paper that was blotted with the subject’s blood); testing included a point of care hemoglobin evaluation (HemoCue, USA) and rapid diagnostic testing (RDT) for malaria (Paracheck Pf, Orchid Biomedical Systems, Goa, India)[32], which were performed in accordance with the manufacturer’s instructions. The DBS were stored refrigerated with a desiccant, pending testing.

Subjects ≥15yrs underwent phlebotomy: 2 EDTA tubes were collected (total 7-10mL whole blood). The blood was processed as follows: one of the tubes was aliquoted directly into cryovials as whole blood. The second tube was centrifuged and used to prepare red blood cell and plasma aliquots. All aliquots were processed the day of sampling and frozen (-18C) pending shipment to the US where testing was conducted. The samples were maintained frozen until testing was initiated.

### Serological testing

#### ELISA (subjects aged >1yr and <15yrs only)

Dried blood spots (DBS) were shipped to Kephera Diagnostics, LLC (Framingham, MA) for antibody testing. Each DBS was eluted with 300ul of kit sample buffer overnight at 4°C. The eluted samples were subsequently tested at 1:100 dilution with an investigational *B*. *microti* ELISA using the kit package insert instructions [33]. The ELISA was configured to detect both IgG and IgM antibodies. For this study, the cut-off defined in the ELISA kit, which was intended for use on serum samples, was modified to equal the mean A_450_ of replicate negative dried blood spot controls plus 3 standard deviations. For purposes of analysis, an S/Co ratio of 1.6 was used as a differentiating criterion, based on a prior study on U.S. blood donors in which donors that were ELISA seroreactive and positive for *B*. *microti* by PCR were found to have S/Co >1.6[33].

#### Indirect fluorescent antibody (IFA) testing (subjects aged ≥15yrs only)

IFA was performed at American Red Cross, Holland laboratory (Rockville, MD) using an approach that has previously been reported[21]. In brief, for the antigen preparation, *B*. *microti*-infected hamster blood (parasitemia 25–60%) were collected in cold anticoagulant (heparin) and washed in phosphate-buffered saline (PBS) to remove plasma and buffy coat. The final pellet was diluted a minimum of 20 times the original blood volume in PBS and added to 12-well IFA slides, 5uL per each well. The slides were first air dried and then dried 2 hours in a 37°C incubator. The slides were stored frozen at -20C°. The animals used for this protocol were female Golden Syrian hamsters purchased from Invigo (Indianapolis, IN). The hamsters were housed by BIOQUAL Inc. (Rockville, MD). Plasma or serum samples were used for testing; the sample was diluted 1 in 64 in PBS; 20 μl was added to each slide well containing the *B*. *microti* antigen and incubated at 37°C for 30 minutes. After incubation, slides were washed in PBS for 10 minutes under agitation, rinsed in distilled water and air-dried. Next, 20 μl of secondary antibody, fluorescein-labeled goat anti-human immunoglobulin G conjugate (ThermoFisher Scientific) was added to each well and incubated at 37°C for 30 minutes and then washed and dried as before. Samples were analyzed by fluorescent microscope at 400X magnification. Positive samples were titered up to 1:1024. Samples initially positive at 1:64 were titered to endpoint.

#### Molecular testing (subjects aged ≥15yrs only)

Testing of whole blood aliquots was performed at Grifols Diagnostic Solutions Inc. (San Diego, CA) using the investigational Procleix *Babesia* assay on the fully automated Procleix Panther system. The assay methodology has been described previously [34]. In brief, the Procleix *Babesia* assay is a Transcription Mediated Amplification-based (TMA), qualitative *in vitro* nucleic acid (RNA) test for the detection of *B*. *microti*, *B*. *divergens*, *B*. *duncani* and *B*. *venatorum* in whole blood specimens. This test was developed with the primary intent of blood donation screening in whole blood lysate pools of up to 16 samples. Whole blood samples are lysed and pooled on the Procleix Xpress system prior to being processed on the Procleix Panther system. The Procleix *Babesia* assay has a demonstrated analytical sensitivity of 1.8 to 3.2 parasites/mL based on the point estimates from probit analysis.

#### Statistical analyses

Presented Mean (Standard Deviation [SD]), Median (Interquartile range [IQR]), and the range of the S/Co signal are presented for the overall sample and stratified by factors of interest such as age and RDT positivity. To test for differences in the S/Co signal between groups the Wilcoxon rank sum test was used. Based on an S/Co ratio of 1.6, households were classified into those that had two or more children with an S/Co ratio greater than 1.6 and those that had one or no child with the high S/Co ratio. To test for the difference in household characteristics between the two groups Fisher’s exact test was used. Room characteristics related to high S/Co ratio in children, factors and household characteristics associated with *Babesia* infection in adults were all evaluated using the Fisher’s exact test. Data were analyzed with STATA version 15.0/IC software (StataCorp, College Station, TX).

#### Outcomes

The outcome of interest is the distribution of sero-reactivity as measured by the ELISA Signal to Cut-off (S/Co) ratio, calculated as the ELISA absorbance of the sample at 450nm divided by the cut-off.

#### Supporting information legends

An open access data set has been made available. The data set include basic demographic data on the participating subjects in addition to questionnaire and household data pertaining to tick exposure, as well as *Babesia* laboratory test results.

## Results

Of the 719 residents (n = 403 in hamlet 119; and 316 in hamlet 483) who were eligible to participate in the study, a total of 570 (79.3%) subjects participated of whom 289 (51%) were female; 271 (48%) were aged ≥1 to <15 years (Table 1). Thirty seven percent of subjects <15yrs were malaria RDT positive as compared to 13% in those 15yrs or older. Reasons for non-participation included absence from the hamlets at the time of examination or refusal. Five children <15yrs were excluded from the analysis given that samples for determination of seroreactivity were not obtained.

**Table 1:** Study population characteristics.

Number of participants	Overall (570 (%))	Hamlet 119 (288(%))	Hamlet 483 (282 (%))
Age group (years)			
<15, n (%)	271 (48)	121 (42)	150 (53)
15–29, n (%)	123 (21)	67 (23)	56 (20)
30–49, n (%)	109 (19)	59 (21)	50 (18)
50+, n (%)	67 (12)	41 (14)	26 (9)
Gender			
Female	289 (51)	134 (47)	155 (55)
Malaria status			
RDT+ (age <15), n (%)	100 (18)	46 (16)	54 (19)
RDT+ (age 15+), n (%)	45 (8)	28 (10)	17 (6)

RDT = rapid diagnostic testing.

### Antibody status in subjects <15yrs

A total of 266 of 271 (98.2%) of those subjects <15yrs old had DBS available for *Babesia* evaluation (Table 2). The signal to cut-off (S/Co) ratios increased by age: the median (Inter Quartile Range [IQR]) and range were 0.94 (0.72, 1.25) in children less than 5 years, and 1.27 (0.94, 1.70) in children >5yrs (p<0.001) (Table 2). With the exception of age, there was not a significant association between the S/Co ratio and gender, the presence of fever and malaria status as ascertained by RDT. In those households with ≥2 children with an S/Co ratio >1.6, there was no association between the S/Co ratio and the material composition of the house or proximity of the house within 10m of an animal pen. There was a suggestive association between crop storage in the house and higher S/Co ratio (Table 3). When comparing those with an S/Co > 1.6 vs. those with an S/Co ≤1.6, the only significant association was that of the number of children sleeping in the room (p = 0.02). All of the households with ≥2 children with S/Co ≥1.6 lived in houses where crops were stored inside. There was no association with sleeping conditions and room characteristics, including the presence of different domestic animals in the rooms, sleeping on animal skins and the presence of bed nets (Table 4).

**Table 2:** Factors associated with ELISA S/Co ratio in children.

	n	ELISA S/Co (mean (SD), median (IQR), range)	Test
Age group (years)			**Wilcoxon rank-sum test P value <0.001**
<5	112	1.05 (0.46), 0.94 (0.72–1.25), 0.38–2.67	
5+	154	1.40 (0.66), 1.27 (0.94–1.70), 0.56–3.82	
Gender			Wilcoxon rank-sum test P value 0.96
Female	133	1.26 (0.62), 1.10 (0.79–1.48), 0.38–3.53	
Male	133	1.25 (0.60), 1.11 (0.83–1.48), 0.49–3.82	
Fever			Wilcoxon rank-sum test P value 0.59
Yes	167	1.29 (0.66), 1.10 (0.80–1.61), 0.45–3.82	
No	99	1.19 (0.52), 1.14 (0.80–1.46), 0.38–3.53	
RDT status			Wilcoxon rank-sum test P value 0.15
Negative	167	1.20 (0.56), 1.10 (0.76–1.48), 0.38–3.82	
Positive	99	1.34 (0.68), 1.14 (0.85–1.52), 0.52–3.53	

ELIS A–enzyme linked immunosorbent assay, SD–standard deviation, IQR–inter quartile range, S/Co ratio–signal to cut off ratio, RDT–rapid diagnostic test

**Table 3:** Household characteristics associated with having two or more children with the S/Co ratio greater than 1.6 in the household.

	Household with one or no children with S/Co ratio >1.6 *n (%)*	Household with 2 or more children with S/Co ratio >1.6 *n (%)*	P value Fisher’s exact
Animal pen within 10 m from home			0.53
Yes	41 (87.2)	6 (12.8)	
No	56 (91.8)	5 (8.2)	
Grass >5 m^2^ from a house			1.0
Yes	28 (90.3)	3 (9.7)	
No	69 (89.6)	8 (10.4)	
Crops stored in a house			0.06
Yes	71 (86.6)	11 (13.4)	
No	26 (100.0)	0 (0.0)	
Roof made of tin or partly tin			1.0
Yes	36 (90.0)	4 (10.0)	
No	61 (89.7)	7 (10.3)	
Windows covered with screens			1.0
Yes	9 (90.0)	1 (10.0)	
No	88 (89.8)	10 (10.2)	
Wall made of bricks			0.52
Yes	39 (92.9)	3 (7.1)	
No	58 (87.9)	8 (12.1)	
Wall made of mud			0.53
Yes	48 (87.3)	7 (12.7)	
No	49 (92.5)	4 (7.6)	
Wall made of sticks			0.76
Yes	41 (91.1)	4 (8.9)	
No	56 (88.9)	7 (11.1)	
Wall made of grass			1.0
Yes	13 (92.9)	1 (7.1)	
No	84 (89.4)	10 (10.6)	

S/C ratio–signal to cut off ratio, n–number, %–percentage.

**Table 4:** Association between the room characteristics where children sleep and having S/Co ratio greater than 1.6.

	Children with S/Co ratio ≤1.6 *n (%)*	Children with S/Co ratio >1.6 *n (%)*	P value Fisher’s exact
Baby cattle in the room			0.31
Yes	3 (60.0)	2 (40.0)	
No	201 (77.9)	57 (22.1)	
Baby goats in the room			0.12
Yes	5 (55.6)	4 (44.4)	
No	199 (78.4)	55 (21.6)	
Poultry in the room			0.59
Yes	42 (75.0)	14 (25.0)	
No	162 (78.3)	45 (21.7)	
Bed nets			0.25
Yes	192 (78.4)	53 (21.6)	
No	12 (66.7)	6 (33.3)	
Bed in the room			0.51
Yes	149 (78.8)	40 (21.2)	
No	55 (74.3)	19 (25.7)	
Anyone sleeps on animal skin			0.88
Yes	76 (76.8)	23 (23.2)	
No	128 (78.0)	36 (22.0)	
Anyone sleeps on mat or cloth			0.51
Yes	145 (76.3)	45 (23.7)	
No	59 (80.8)	14 (19.2)	
Number of people sleep in a room			0.02
1–2	44 (66.7)	22 (33.3)	
3–4	118 (83.7)	23 (16.3)	
5–7	42 (75.0)	14 (25.0)	
Number of animals sleeping in a room			0.31
0	158 (79.0)	42 (21.0)	
1	46 (71.9)	18 (28.1)	
2	2 (66.7)	1 (33.3)	

S/C ratio–signal to cut off ratio, n–number, %–percentage.

### Antibody status in adults

A total of 291 subjects ≥15yrs contributed samples that were tested by IFA for antibodies against *B*. *microti*, four (1.4%) of whom were IFA reactive (two each at titers of 128 and 256). A further 28 (9.6%) were inconclusive. There was a significant association with older age (p = 0.006); there were no significant associations between IFA positivity and gender, fever or malaria status (i.e. as ascertained by RDT) (Table 5). Two of the IFA positive subjects reported a history of malaria in the preceding 6-months; two did not. No risk factors were significantly associated with antibody status in adults, although there was again a suggestion of increased risk with crops stored inside the house; all of the antibody positive adults had crops stored inside the house (Table 6).

**Table 5:** Factors associated with *Babesia* infection in adults.

Babesia status	Negative *n (%)*	Positive *n (%)*	Inconclusive *n (%)*	P value Fisher’s exact
Age group (years)				**0.006**
15–29	113 (95.8)	1 (0.8)	4 (3.4)	
30–49	93 (86.9)	1 (0.9)	13 (12.2)	
50+	53 (80.3)	2 (3.0)	11 (16.7)	
Gender				0.26
Female	136 (91.9)	1 (0.7)	11 (7.4)	
Male	123 (86.0)	3 (2.1)	17 (11.9)	
Fever				0.40
Yes	143 (87.2)	2 (1.2)	19 (11.6)	
No	115 (91.3)	2 (1.6)	9 (7.1)	
RDT status				1.00
Negative	218 (88.6)	4 (1.6)	24 (9.8)	
Positive	41 (91.1)	0 (0.0)	4 (8.9)	

n–number, %–percentage, RDT–rapid diagnostic test.

**Table 6:** Household characteristics associated with *Babesia* infection in adults.

	Negative *n (%)*	Positive *n (%)*	Inconclusive *n (%)*	P value Fisher’s exact
Animal pen within 10 m from home				0.30
Yes	45 (79.0)	0 (0.0)	12 (21.0)	
No	72 (79.1)	4 (4.4)	15 (16.5)	
Grass >5 m^2^ from a house				0.07
Yes	25 (69.4)	0 (0.0)	11 (30.6)	
No	92 (82.1)	4 (3.6)	16 (14.3)	
Crops stored in a house				0.24
Yes	84 (75.7)	4 (3.6)	23 (20.7)	
No	32 (88.9)	0 (0.0)	4 (11.1)	
Roof made of tin or partly tin				0.72
Yes	51 (82.3)	1 (1.6)	10 (16.1)	
No	66 (76.7)	3 (3.5)	17 (19.8)	
Windows covered with screens				1.00
Yes	11 (84.6)	0 (0.0)	2 (15.4)	
No	106 (78.5)	4 (3.0)	25 (18.5)	
Wall made of bricks				1.00
Yes	50 (78.1)	2 (3.1)	12 (18.8)	
No	67 (79.8)	2 (2.4)	15 (17.9)	
Wall made of mud				0.46
Yes	54 (75.0)	2 (2.8)	16 (2.2)	
No	63 (82.9)	2 (2.6)	11 (14.5)	
Wall made of sticks				0.43
Yes	47 (85.5)	1 (1.8)	7 (12.7)	
No	70 (75.3)	3 (3.2)	20 (21.5)	
Wall made of grass				0.45
Yes	12 (80.0)	1 (6.7)	2 (13.3)	
No	105 (79.0)	3 (2.2)	25 (18.8)	

n–number, %–percentage.

### Test for infection in subjects aged ≥15yrs

None of the TMA test results were reactive.

## Discussion

Findings from our study suggest that *B*. *microti* is present in the two surveyed African communities. However, given the low observed rate of seroreactivity, its clinical impact in these communities is uncertain and potentially low. At least in those aged 15 or older, none had evidence of active parasitemia as reflected by molecular (TMA) testing for *Babesia* infection. The low numbers of seroreactive individuals is a constraint that may have impacted the findings. Larger sample sizes would be needed to ascertain true prevalence of exposure. While none of the postulated risk factors for exposure were statistically significantly associated with antibody positivity, there is a strong suggestion that storing crops, in this case, maize and millet, inside the houses might lead to greater exposure. While plausible that ticks, which have attached themselves to crops or their associated plant debris, could place residents at risk of tick bites and associated *Babesia* transmission, this needs to be interpreted in the context of multiple negative risk factors. The study also revisits the challenges of investigational study of a neglected pathogen.

The study offers further support for the occurrence of *Babesia* infection in humans in Africa. Evidence for this is as follows: first, there were individuals with high S/Co ratios in the younger age group and there was also an association between seroreactivity (based on an assigned cutoff) and age, which is to be expected. We also found four seroreactive cases in the older “adult” age group at modest titers (128 and 256); this rate (1.4%) of seroreactivity (as ascertained by IFA) is not dissimilar from that in established, high endemic areas such as those in the United States [35]. The absence of molecular reactivity (where evaluation was restricted to the older age group) suggests that at the time of the study, there were no active infections. This was not surprising: although a molecular result is a better correlate of active parasitemia, only about 8–20% of seroreactive individuals are expected to have a NAT reactive result [5, 33, 36]. Furthermore, this was a surveillance study of local residents rather than targeted assessment of acutely ill individuals. Not unique to this study, correlation between seroreactivity and molecular reactivity is poorly defined [37].

Unlike the preceding pilot study [23], the seroreactivity–at least in the younger group of subjects- was not associated with RDT positivity for malaria. While the reasons for this difference are not entirely clear, there are several possible explanations. First, the pilot study covered a much broader range of populations (villages/hamlets) than the present study. The latter was limited to only two hamlets, whereby differences in the populations may have contributed to the observation (or lack thereof). Second, the relationship between S/Co and malaria RDT result may have been correlative rather than causal (i.e. perhaps similar risk factors contributed to both results), confounding the true causes for each finding in the study populations. Third, stochastic effect cannot be definitively excluded, particularly given the modest number of samples. Other factors that were considered include the specificity of the RDT. The RDT that was used targets histidine-rich protein 2 (HRP-2), which is specific for *P*. *falciparum*. The assay is well established, having been used in multiple studies, both in Tanzania[38–40] as well as regionally[41, 42]. While sensitivity of the assay is reportedly high (90–100%), its specificity has been more variable (52–99.5%)[38, 43]. It is uncertain to what extent this impacted the findings. There still remains the possibility of cross-reactivity with other pathogens, including other *Babesia* species such as *B*. *bigemina* and *B*. *bovis* [29, 30] and parasites that have been reported (e.g. *Entopolypoides macaci* [44], *Theileria* [45] regionally. Independent of whether showing zoonotic potential, it is plausible that exposure to these other parasites can confound the serologic findings for *B*. *microti*.

This study has limitations. First, the testing approach was not uniform across the study population. We elected to conduct minimally invasive sampling in those aged 15 or under. Consequently, only DBS were available for this group, precluding head to head comparison with the older group of subjects in which formal blood sampling was undertaken. The larger volume in the older age group allowed for IFA and molecular assessment. In hindsight, a better approach might have been to apply the same test (ELISA) to all subjects. Second, as described in the preceding pilot study, the *B*. *microti* ELISA had not been validated for this population or setting [23]; instead it had been developed for blood donor screening for antibodies against *B*. *microti* in the US using plasma or serum samples. In this case, the testing protocol was modified to allow DBS testing. In addition to differences in test performance, the environment imposes its own challenges, whereby the possibility of false positivity and/or cross-reactivity with other local pathogens merits consideration. Similarly, IFA cut offs have not been developed for this our study population; in the US, the CDC recommends a titer of ≥ 256 (or ≥ 64 in epidemiologically linked blood donors or recipients) for surveillance [46]. Further, there are no data that compare the actual IFA assay that was used in this study to the ELISA. Nonetheless, during the ELISA validation there was a reported concordance between EIA and IFA of 99.34%[8]. Further, in a subsequent study, 91% of IFA-positive clinical babesiosis patients were ELISA positive [33]. The caveat is that the current study did not select for individuals with clinical babesiosis. As such, the expected level of concordance is uncertain. Third, there isn’t a gold standard (i.e. reference) test for *Babesia*. Each assay offers support for or against diagnosis, yet alone fails to address the diagnostic uncertainty. As a neglected pathogen, reference standards are somewhat incomplete and most data pertaining to diversity of human *Babesia* isolates have been regionally focused (i.e. in the US) [47] and may not be applicable to the target location. While the preceding pilot study lend credence to the findings, true prevalence data are lacking. We acknowledge that the positive predictive value of the individual assays in this setting is unknown. As an exploratory endeavor, we tried to compensate for any confounding interference by adjusting cut-offs accordingly. For example, for the interpretation of risk in the context of the ELISA results, a conservative, provisional S/Co ratio of 1.6 was applied. The S/Co of 1.6 was derived from a previous validation study on U.S. blood donors in which all seroreactive subjects who were also PCR positive exhibited an S/Co > 1.6 [33, 37]. This is not to say that PCR or similar molecular reactivity is a gold standard; rather a higher S/Co ratio reflects a stringent threshold for exposure with or without active parasitemia. In our study, the absence of TMA reactivity offers convincing evidence that none of the subjects in the older group were actively infected with the four species targeted by the Procleix Babesia assay. Fourth, without a complete travel history one cannot be certain that exposure to *B*. *microti* occurred outside of the two hamlets. Finally, to echo conclusions from the preceding pilot study, multidisciplinary input, with insight into the local entomology and veterinary input, would help to contextualize the findings by assessing the ecological suitability of the target environment for supporting tick exposure. While zoonotic *Babesia* spp. have been isolated from ticks in Africa (e.g. Nigeria)[28], *B*. *microti*—specifically—has not yet been recovered from regional ticks. As such the putative tick vector is unknown. Furthermore, high homology (97.9% sequence identity) between *B*. *microti* and *E*. *macaci*, could impede the ability of existing nucleic acid or serologic assays to distinguish the two parasites [44]. There is good evidence of regional Babesiosis in domestic and animal populations. This does not necessarily translate to human risk.

There is value to surveillance of *Babesia* in novel populations. This is particularly the case in Africa and other settings where malaria is endemic. Febrile illness in low resource settings is all too frequently treated empirically as malaria, without laboratory confirmation. Such ignores a broad differential diagnosis. In one study in Northern Tanzania, 60.7% of subjects who presented with fever were diagnosed with malaria yet only 1.6% of cases were ultimately confirmed as having malaria [48]. While empiric treatment likely stems from local resource constraints, specifically a lack of capacity for laboratory investigation [49], there are fundamental problems with this strategy. Misdiagnosis and treatment delays may have serious if not fatal consequence, while inappropriate antimicrobial therapy also risks treatment failure and development of resistance, broadly detracting from its effectiveness across a range of pathologies.

In conclusion, our study further supports the notion that *B*. *microti* is encountered locally in Tanzania, at least in two communities in Kilosa, district. The study’s findings raise questions about the clinical significance of *Babesia* infection (at least *B*. *microti*) in this setting yet confirms the value of exploratory investigations (including in Africa). Pilot investigation has yielded unexpectedly high prevalence of *Babesia* in other populations [17]. Where there is active infection, misdiagnosis of babesiosis has serious clinical ramifications that might otherwise be avoided through timely intervention. This further argues for rapid, point of care diagnostic tools to expand surveillance in remote or low-resourced settings.

## Supporting information

S1 Babesia data set (CSV)

S1 Variable labels (DOCX)
