# Supplementary material for: The Babesia observational antibody (BAOBAB) study: A cross-sectional evaluation of Babesia in two communities in Kilosa district, Tanzania
Source: PLoS Negl Trop Dis. 2019 Aug 14;13(8):e0007632. doi: 10.1371/journal.pntd.0007632 (PMC6693791; doi:10.1371/journal.pntd.0007632)
Supplement: S1 Variable labels — (DOCX) [file pntd.0007632.s002.docx]

**Variable labels**

---------------------------------------------------------------------------------

variable name variable label

---------------------------------------------------------------------------------

studyID Unique study participants ID

hamlet Village ID

age Age in years

gender Gender:1=male,2=female

bab_RDTresult result of malaria rapid test:1=positive, 9=treatment

needed,2=negative,3=undetermined

fever Have you had fever in the last 3 months?:1=yes,2=no,9=don’t know

s_co Signal to cut off ratio for babesia testing of children

results_bab Babesia status for adults

results_pm Plasmodium molecular results

bcattle in this room are there baby cattle?:1=yes,2=no

bgoats in this room are there baby goats?:1=yes,2=no

Rpoultry in this room is there poultry?:1=yes,2=no

bedNet do they sleep in a bed net?:1=yes,2=no

bed is there a bed?:1=yes,2=no

animalSkins does anyone sleep on animal skin?:1=yes,2=no

mats does anyone sleep on mats or cloth?:1=yes,2=no

num_people Number of individuals sleeping in one room

num_animals Number of animals sleeping in a given room

---------------------------------------------------------------------------------
